# Information ranks highest: Expectations of female adolescents with a rare genital malformation towards health care services

**DOI:** 10.1371/journal.pone.0174031

**Published:** 2017-04-20

**Authors:** Elisabeth Simoes, Alexander N. Sokolov, Andrea Kronenthaler, Hanna Hiltner, Norbert Schaeffeler, Katharina Rall, Esther Ueding, Monika A. Rieger, Anke Wagner, Leonie S. Poesch, Marie-Christin Baur, Judith Kittel, Sara Y. Brucker

**Affiliations:** 1 Women’s Health Research Institute, Department of Women’s Health, University Hospital Tübingen, Tübingen, Germany; 2 Staff Unit of Social Medicine, Eberhard Karls University of Tübingen Medical School and University Hospital Tübingen, Tübingen, Germany; 3 Institute of Sociology, Faculty of Economics and Social Sciences Eberhard Karls University of Tübingen, Tübingen, Germany; 4 Psychosomatic Medicine and Psychotherapy, University Hospital Tübingen, Tübingen, Germany; 5 Institute of Occupational and Social Medicine, and Health Services Research, University Hospital Tübingen, Tübingen, Germany; Tilburg University, NETHERLANDS

## Abstract

**Background:**

Access to highly specialized health care services and support to meet the patient’s specific needs is critical for health outcome, especially during age-related transitions within the health care system such as with adolescents entering adult medicine. Being affected by an orphan disease complicates the situation in several important respects. Long distances to dedicated institutions and scarcity of knowledge, even among medical doctors, may present major obstacles for proper access to health care services and health chances. This study is part of the BMBF funded TransCareO project examining in a mixed-method design health care provisional deficits, preferences, and barriers in health care access as perceived by female adolescents affected by the Mayer-Rokitansky-Küster-Hauser syndrome (MRKHS), a rare (orphan) genital malformation.

**Methods:**

Prior to a communicative validation workshop, critical elements of MRKHS related care and support (items) were identified in interviews with MRKHS patients. During the subsequent workshop, 87 persons involved in health care and support for MRKHS were asked to rate the items using a 7-point Likert scale (7, strongly agree; 1, strongly disagree) as to 1) the elements’ potential importance (i.e., health care expected to be “best practice”, or priority) and 2) the presently experienced care. A gap score between the two was computed highlighting fields of action. Items were arranged into ten separate questionnaires representing domains of care and support (e.g., online-portal, patient participation). Within each domain, several items addressed various aspects of “information” and “access”. Here, we present the outcome of items’ evaluation by patients (attended, *N*_PAT_ = 35; respondents, *N*_RESP_ = 19).

**Results:**

Highest priority scores occurred for domains “Online-Portal”, “Patient participation”, and “Tailored informational offers”, characterizing them as extremely important for the perception as best practice. Highest gap scores yielded domains “Tailored informational offers”, reflecting perceived lack of disease-related information for affected persons, medical experts, and health insurance companies, “Online-Portal” (with limited information available on specialist clinics and specialized doctors), and regarding insufficient support offers (e.g., in school and occupational settings). Conversely, lowest gap scores were found with group offers for MRKHS patients (“Transition programs”) and MRKHS self-help days (“Patient participation”), suggesting satisfaction or good solutions in place.

**Discussion:**

The importance assigned to disease-related information indicates that informational deficits are perceived by patients as barriers, hindering proper access to health care, especially in an orphan disease. Access to health-related information plays a role for all persons seeking help and care. However, the overwhelmingly high scores attributed to these elements in the context of an orphan disease reveal that here improved information policies are crucial, demanding for institutionalized solutions supported by the health care system.

**Implications for practice:**

The disparity between experience of care and attribution as best practice detected describes areas of action in all domains involved, highlighting information related fields. New concepts and structures for health care in orphan diseases could draw upon these patient-oriented results a) regarding orphan-disease specific elements demanding institutionalized reimbursement, b) essential elements for center care and corresponding networks, and c) elements reflecting patients´ participation in the conception of centers for rare diseases.

## Introduction

The probability of utilizing health care services largely depends on the balance between individuals’ perceptions of their needs and their attitudes, their health literacy, beliefs, and previous experience with health services. In many countries, therefore, policy responses have shifted from attempts to change patients´ behaviors to acknowledging patients´ needs and managing their demands through developing a graded service (e.g., [1]). Health care for adolescents faces particular demands, especially for the growing and significant number of young persons who are entering adulthood with ongoing needs of support in order to unfold their physical, social and psychological potential [2]. This superposition of problem areas calls for a thorough assessment of needs and preferences, which informs a proper design of future health care.

### Transitional care

Age-related transitions are periods, in which people with specific medical care needs transfer from the health care settings targeting their current age group to the settings targeting the next group. One of the most prominent transitions occurs in adolescence: it lasts, according to the World Health Organization, WHO, definition [3], from 10 through 19 years of age. During this time, young people with particular needs in medical care gradually transfer from the child-centered to the adult-centered medicine. The transition represents a multidimensional, multidisciplinary, and active process, targeting the physiological, medical, psychosocial, and educational needs of young people [4]. Additionally, adolescents in general have unique barriers to access appropriate health care [5]. An important and often overlooked factor in adolescents’ access to care is that commonly, they do not have an adult person to help them navigate the increasingly complicated health care system. This fact is calling for assistance and guidance, especially in case of an orphan disease.

### Health care issues in orphan diseases

Being affected by an orphan disease complicates the situation in several important respects. Transition into the adult life represents profound psychobiological and social change for all young people and their families. However, it is even more demanding for adolescents with long-term care needs and a rare disorder: The transition is largely complicated by transforming adolescents’ needs and especially difficulties in *access* to proper medical and appropriate (psycho)social services. Disorders, in which transition is particularly important, include complex genital malformations that are rare (orphan) diseases. A disease is considered rare, if less than 5 to 10 per 10,000 people are affected. Over 7,000 of the 30,000 known diseases represent orphan diseases, and altogether they affect many persons: Only in Germany, for instance, live about four millions patients [6]. The incidence of the most common genital disorder in females, the Mayer-Rokitansky-Küster-Hauser syndrome (MRKHS, which is characterized by congenital missing of uterus, cervix, and upper two thirds of the vagina) is estimated by 1:4,000 to 5,000 female life births [7, 8], or 1:14,000 up to 1:50,000 of all women in the population. The condition often yields a considerable burden for the patients and their families [8]. When dealing with a single orphan disease, one does not address large patient populations but faces instead with a multitude of specific challenges. Only recently, however, health care systems have become more alert to the central role of access and barriers as risks for good outcome both at the individual and the national levels [9].

Health care in orphan diseases is still characterized by tedious patient careers and deficits of care and support. Overall scarcity of services in orphan diseases may partly account for the lengthy and uneasy patient careers; however, this also suggests a lack of proper management and integration of care. As a result during the transition period, a large fraction of young people with enduring needs become detached from the care system [10] with clear negative consequences for both the individual patient and the entire health care system as both face with substantial burden and costs due to the sequelae. Long distances to dedicated institutions and scarcity of knowledge even among medical doctors, may present major obstacles for proper access to health care services.

At present, a diagnosis of female genital malformations is made at various points in time depending on the disorder and ranging from birth through adulthood, but very often too late, with a tedious patient career full of wrong diagnostic and treatment attempts (40.9%; [11]). Frequently, diagnostic attempts take place during the transition period. Importantly, genital disorders, in addition to the (psycho)social and personal concerns, strongly modulate the adolescent’s sexual development. In all these respects genital malformations can be seen as a model for transition issues in orphan diseases: The problems of the transition period as outlined above, deficits in access to medical care, its discontinuity, lacking integration and social support for development may become even more prominent than in non-orphan disorders.

The German Advisory Council on the Assessment of Developments in the Health Care System released its special report 2009 based on the analysis of international evidence, local German projects, and research literature [12]. The report revealed deficits in care during the transition periods, largely linking them to the weaknesses in coordination. The available sources did not allow reliable conclusions to be made as to the extent of over-, under-, and misdirected provision of care as well as the specific expectations of affected individuals who beyond medical treatment, may be in need of support regarding other puberty-related issues such as sexuality, separation from the parental home, social integration, and career planning [13].

### Dimensions of access

Publications addressing the role of access have increased in the international literature since the times of neglecting its basic relevance to medical outcomes and safety culture. Similar to “quality”, “access” is a complex concept that can be assessed on several dimensions [14, 15]. If services are available and there is an adequate supply of services, then the opportunity exists to obtain health care, and patients may “*have access*” to services. The extent, to which patients “*gain access*” however, depends on additional factors, as financial, organizational and social or cultural barriers may limit the utilization of services. Thus, access measured in terms of utilization is dependent on the affordability, physical accessibility, and acceptability of services rather than merely on adequacy of supply. If available services are relevant and effective as promised by the conception of centers [15], patients can “*gain access to satisfactory health outcomes*”.

*Both availability of services* and *barriers to access* them have to be considered in the context of the differing perspectives, health needs, and socioeconomic and cultural settings of diverse population groups in the society and distinct patient groups. Rare diseases hereby require special attention. Proof of access is use of service, not only mere presence of a facility [16]. *Financial barriers* influence the patient´s utilization of services even in the nations, in which like in Germany, in spite of the essentially “free” system at the point of use to most of the population due to the obligatory health insurance, charges still may apply for special services. The impact of user charges and other costs of accessing care affect different socioeconomic groups in different ways. *Organizational barriers* to access may result from failure to design services around the needs of patients. Access to highly specialized health care services and proper support that meet the patient’s specific needs are crucial for health outcome, especially during age-related transitions within the health care system.

### Finding the right way through the system has become a major challenge

The ultimate objective of any health care system is to promote or preserve health [15]. The US Institute of Medicine defines access as the “timely use of personal health services to achieve the best possible outcome” [16]. On this dimension, access can be measured using appropriate indicators of health status. For example, organizational barriers to access may result in delays in treatment and may lead to worse clinical outcomes. Assessment of access according to health outcomes, rather than according to availability or utilization of services may alter conclusions. *A concern to ensure* that health care resources are mobilized to meet the needs of different groups in the population is central to the concept of access.

A tight cooperation of professionals from multiple medical and social disciplines is required for successful diagnosis, treatment, and research on orphan diseases. Both health care facilities that largely vary by region and socioeconomic disparities demand the development of tailored concepts and new provisional structures, especially for young patients with rare diseases such as those with a rare genital malformation reported in this study. Considerable scope remains for tailoring targeted offers for these patients during the transition period in order to reduce weaknesses in coordination within the health care system and avoid inequity in health chances.

The present study is part of the *BMBF-funded TransCareO research project* with the aim to explore which targeted and coordinated provision of care, structural components and support would be appropriate to improve transitional care for female adolescents with MRKHS, an orphan disease described in more detail elsewhere [17]. The entire project focuses on needs, preferences, and perspectives of the affected individuals and the different groups involved in their care and support. In this context, the present study deals with determining the importance attributed to specific issues and structures identified as deficits and needs and mirroring attitudes and preferences expressed by the affected persons (patients) and their experience with current health care offers. These results significantly contribute to priority setting for and shaping of future health care offers for female adolescents with MRKHS, other rare diseases, and genital malformations or injuries. To the best of our knowledge, no previous study has dealt with determining such preferences including access and the role of information in accessibility of health and supportive care services with regard to MRKHS *as an orphan disease* within either German or any other national health care system.

The work was conducted at the Center for Rare Female Genital Malformations (*Zentrum für Seltene Genitale Fehlbildungen der Frau*, *ZSGF*) that is an integral part of the University Women’s Hospital, Department of Women´s Health, Tübingen, and unique of its kind in Germany. The center has a longstanding specialization and experience in treating genital disorders in females. The treatment is offered by specialists in pathology, human genetics, psychosomatic medicine, endocrinology, and the pediatric department. Together they have been striving for continuous improvement of their treatment offers and support for this particular group of young females. Based on this outstanding expertise, this project has aimed to add to the center structure a concept for integration and networking, meeting the challenges of proper targeting and avoiding inequity in health chances, providing accessibility of health care provision, and tailored information in a changing society [14] to those who would not readily find her way to the specialist care needed.

## Methods

The reported study is part of a multi-method project for the development of a provisional model for female adolescents with *Mayer-Rokitansky-Küster-Hauser syndrome* (MRKHS) as an orphan disease, which is described in more detail elsewhere [17]. The present work reports the findings on patients’ expectations and preferences obtained in a workshop setting (Open Space Technology [18]) for validating the results of preceding interviews. *Communicative (or dialogical) validation* as a methodological step comprises sharing the researchers´ understanding and interpretation of the data with the participants and interviewees to ensure that the researchers correctly interpret and analyze the participants’ response [18]. Communicative validation adds value to the rigor of interview data analysis, likely promoting greater research insights. The study was approved by the Ethics Committee at the Eberhard Karls University of Tübingen and the University Hospital Tübingen and conducted according to the principles expressed in the Declaration of Helsinki. Informed written consent was obtained from all participants with participants of minor age, from the next of kin, caretakers, or guardians. The data were collected and analyzed anonymously.

### Participants

This study reports the data of female patients with MRKHS who attended the communicative validation workshop. All persons who had taken part in the preceding interviews, and the interested public, including medical professionals, were invited to attend the workshop. Specifically, invitations were sent out to about 500 persons of the following groups: present and previous MRKHS patients of the Department of Women´s Health, Tübingen, persons who previously took part in the Department’s self-help workshops on MRKHS, all persons actively taken part in the project at previous stages by contributing in an interview or questionnaire, persons with special competence either in a medical specialty, health system, health insurance, or the educational system. The invitation included the possibility of introducing further persons (e.g., parents, a friend) to the workshop. A total of 87 persons followed the invitation, including 35 female patients in the age range of 10–19 years at the time of diagnosis (*N*_PAT_ = 35). This study reports the data of patient respondents (*N*_RESP_ = 19) who returned their questionnaires during the workshop.

### Elements of health care

Prior to the communicative validation workshop, critical elements (items) of MRKHS related care and support were identified as deficits and needs during interviews run with the affected adolescents and women. For presentation purposes at the workshop, the items obtained during the preceding interviews were assigned to a total of ten domains of health care provision and support, with each domain listed in a separate questionnaire. The domains comprised ten to 23 distinct items. A total of 200 items were presented for rating with 50 elements addressing the topic *access*. They were presented in different working groups and thematic frameworks in order to reduce contextual bias. This study reports the outcome for the domains “Online-platform” (23 items), “Patients´ participation” (23 items), “Tailored informational offers” (18 items), “Needs of and offers to parents and relatives” (11 items), and “Transitional programs” (ten items) as assessed by the affected adolescents (MRKHS patients). The domains reported here were chosen due to their close relation to the proper access to health care services. The communicative validation workshop took place prior to the construction of the tentative model for health care provision and support in MRKHS [17].

### Procedure

Upon arrival to the workshop, in an input session in the plenum the participants received information regarding the results of the preceding interviews and the crucial elements of care identified. The results were also condensed in poster presentations as basic information for the working groups. Afterwards the participants were asked to choose a working group from those offered according to the established domains. The participants were also free to open an additional working group by themselves (“Open Space” [18]). According to the communicative validation demands, the elements of care and support that were deemed important during the preceding interviews were discussed in these working groups. The participants were free to change the groups. Finally, each participant rated the elements presented in the questionnaires. All participants were asked to rate elements of all domains as a report summarizing each working group´s discussion and results was made public by fixing it to a “news wall” and the results of all working groups were presented in the plenum.

### Gap score

All elements of provisional and supportive services deemed important and/or deficient during previous interviews and/or by analysis of literature were listed in separate domain-specific questionnaires. Using a 7-point Likert scale (1, “strongly disagree” through 7, “strongly agree”), workshop participants were asked to rate the extent to which each item represented either best practice (i.e., an item “is very important for a good care”; priority) or current care (i.e., an item “is implemented in the current care“; see examples below, Tables 1 and 2, and S1–S5 Tables). Anticipated satisfaction with each item is conceived as the difference, i.e. a “gap” score, between the “best practice”, or priority, and the experienced “current care” scores [19]. The gap score informs therefore about expectations, preferences and importance of single elements as expressed by the different participant groups (patients, parents, medical experts, and other interested persons), representing distinct points of view on MRKHS care. The results will be used for a final tuning of the model for MRKHS care provision. At a later stage of model implementation, the rating procedure may be repeated to inform about satisfaction with care provision according to the model. For example, in a study evaluating the impact of a coordinated transitional care program for adolescents with juvenile idiopathic arthritis, gap scores proved a reliable tool to inform about progress with health care services [19, 20].

**Table 1:** “Tailored information offers” domain items listed according to their gap and priority scores (for original German version, see S4 Table). The letter (here, F) codes the questionnaire domain and the number (1 to 18), the item’s running position in the questionnaire. Each item had to be ranked using a 7-point scale (1, *strongly disagree*, through 7, *strongly agree*) on two occasions (as to both actual and target, i.e., best practice, state of care).

Item	Score	Item content
F1	7	Illustrated information material (e.g., as to the surgery options) **is / are very important for a good care** ||**. . .is / are implemented in the current care**
F2	7	A medical information flyer on MRKHS for after-care professionals with contact persons in the specialized treatment center [..]
F3	7	Information on specialized treatment centers, contact persons, etc. [..]
F4	7	An information booklet on psychological counseling and therapy (e.g., with addresses of specialized psychotherapists) [..]
F7	7	Information on self-help networks and fora [..]
F9	7	A flyer on the disease (e.g., What is MRKHS?) [..]
F10	7	An information booklet on health insurance in MRKHS (e.g., regulations as to the surgery and travel expenses, statutory health insurance services, private health insurance, and severely handicapped pass for MRKHS) [..]
F11	7	For medical experts: an MRKHS flyer with professional information (such as treatment course: diagnose–surgery–after-care–specialized treatment centers and contact persons; assistance with prescriptions of crèmes, functional pants, etc. [..]
F12	7	For medical experts: an MRKHS flyer with professional information on differential diagnosis (e.g., in the absence of menorrhea) [..]
F13	7	A flyer for health insurance companies on MRKHS and the therapies (e.g., types of surgery, prescriptions of multiple functional pants, phantoms, and crèmes) [..]
F14	7	For health insurance companies: a case management instruction (e.g., internal training of „experts for rare diseases”in the companies) [..]
F16	7	Introduction and awareness for MRKHS and other genital malformations in the school, e.g., in biology classes (extension of school curricula) [..]
F8	6.5	A FAQ flyer covering the issues concerning with MRKHS [..]
F15	6	For The Federal Joint Committee (G-BA, the highest decision-making body of the joint self-government of physicians, dentists, hospitals and health insurance funds in Germany): information for justification of special arrangements and regulations for patients with rare diseases (e.g., special outpatient care budget, quality certified treatment centers, extended general practitioner care, auxiliary aid directory) [..]
F5	5.5	An information booklet on supervision offers, coaching [..]
F6	5	Information on advisory services for occupational training, advanced education, and career [..]
F17	5	A training offer from treatment centers for interested teachers on MRKHS and rare genital malformations, and on their teaching in the school [..]
F18	4	An advanced training on rare diseases and, in particular, MRKHS for guidance counselors in the school [..]

**Table 2:** “Online-Portal” domain items listed according to their gap and priority scores (for original German version, see S5 Table). **”** The letter (D) codes the questionnaire domain and the number (1 to 23), the item’s running position in the questionnaire. Each item had to be ranked using a 7-point scale (1, *strongly disagree*, through 7, *strongly agree*) on two occasions (as to both actual and target, i.e., best practice, state of care).

Item	Score	Item content
D1	7	Information webpages on the treatment center internet platform tailored to specific queries and needs of all groups involved–patients, parents, siblings, partners, physicians in private practice, etc. **is / are very important for a good care** ||**. . .is / are implemented in the current care**
D2	7	That the confidentiality of (forum) participants will be secured through partly open and partly protected domains on the treatment center web presence [..]
D3	7	Confidential (protected) query portals and discussion for all groups involved [..]
D4	7	That the treatment center internet platform will offer answers to frequently asked questions (FAQ) [..]
D5	7	Detailed (including background) information on MRKHS on the treatment center internet platform [..]
D6	7	Information on the treatment center internet platform as to other hospitals and physicians in private practice specialized in MRKHS (who endorsed their disclosure) [..]
D7	7	Information on the contact addresses on the treatment center internet platform [..]
D8	7	Information on choice of therapy and, in particular, surgery options on the treatment center internet platform [..]
D9	7	Information on the state-of-the-art in MRKHS research (e.g., uterus transplantation) on the treatment center internet platform [..]
D10	7	Adoption information on the treatment center internet platform [..]
D11	7	Information on the weblinks to the specialized TV-broadcasts on the treatment center internet platform [..]
D12	7	That information booklets and flyers can be downloaded from the treatment center internet platform as pdf files (e.g., as support for general practitioners) [..]
D14	7	That weblinks exist between information platforms themselves or by way of the treatment center [..]
D15	7	That the treatment center web presence will be readily found on the internet, straightforward, accessible, and easily comprehendible [..]
D16	7	That the treatment center web presence will be maintained by a full-time administrator for securing its sustainable updates [..]
D17	7	That the MRKHS fora will be moderated and monitored [..]
D18	7	That experts swiftly and reliably respond to the queries posted on the treatment center internet platform [..]
D20	7	Up-to-date information as to self-help group meetings on the treatment center internet platform [..]
D21	7	Up-to-date information as to conferences and advanced training events on MRKHS on the treatment center internet platform [..]
D22	7	Weblinks to cooperating health insurance companies on payment information (such as acquisition of additional functional pants, phantoms) on the treatment center internet platform [..]
D13	6	That the design of treatment center information platform will comprise both a central domain and dedicated subdomains (e.g., on specific topics, for particular groups) [..]
D19	6	That the authorized center experts will have time budget dedicated specifically to their work on the center internet platform [..]
D23	6	An information booklet on health insurance payments in MRKHS such as regulations for surgery and travel expenses, statutory health insurance payments, private health insurance and MRKHS, severely handicapped pass [..]

### Statistical analysis

The individual scores obtained were not distributed normally as assessed by the Shapiro-Wilk test, and therefore were processed using non-parametric statistics. For each item, we computed median scores and their 95% confidence intervals (CI). Further, a gap score for each item was calculated as a difference between the “best practice” (“Target”) and the experienced “current care” (“Actual”) responses, revealing both accomplishments and possible deficits in the present state of MRKHS care. The pairwise differences between the scores for dependent samples were then evaluated using the non-directional (two-tailed) Wilcoxon signed-rank *W* tests.

## Results

### Elements of care

The elements of care, assigned to the distinct domains, mirror the essential aspects of health care obtained, i.e. perceived deficits, needs, and items for operationalization. The items are presented in detail in Tables 1 and 2 for the domains “Tailored information offers” and “Online-Portal”, and for other three domains, in S1–S3 Tables.

### Ranking outcome

**Fig 1:**
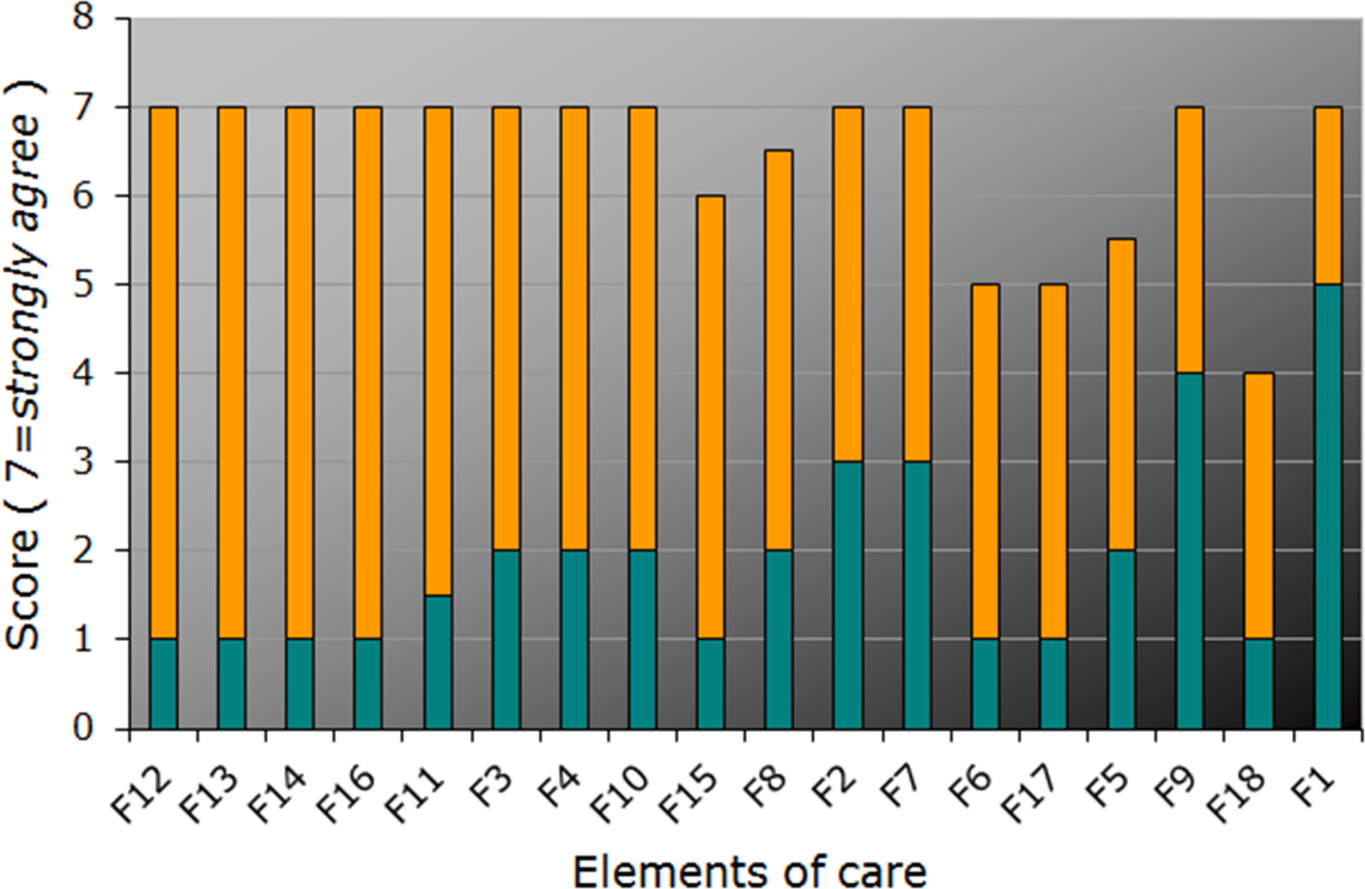
Priority score (*best practice* / “Target”, *full bar height*), current care score (“Actual”, *turquois*), and gap score, *orange*, for items from the “Tailored information offers” domain, ordered according to the gap and priority scores (for item descriptions, see Table 1). Each bar represents median scores.

**Fig 2:**
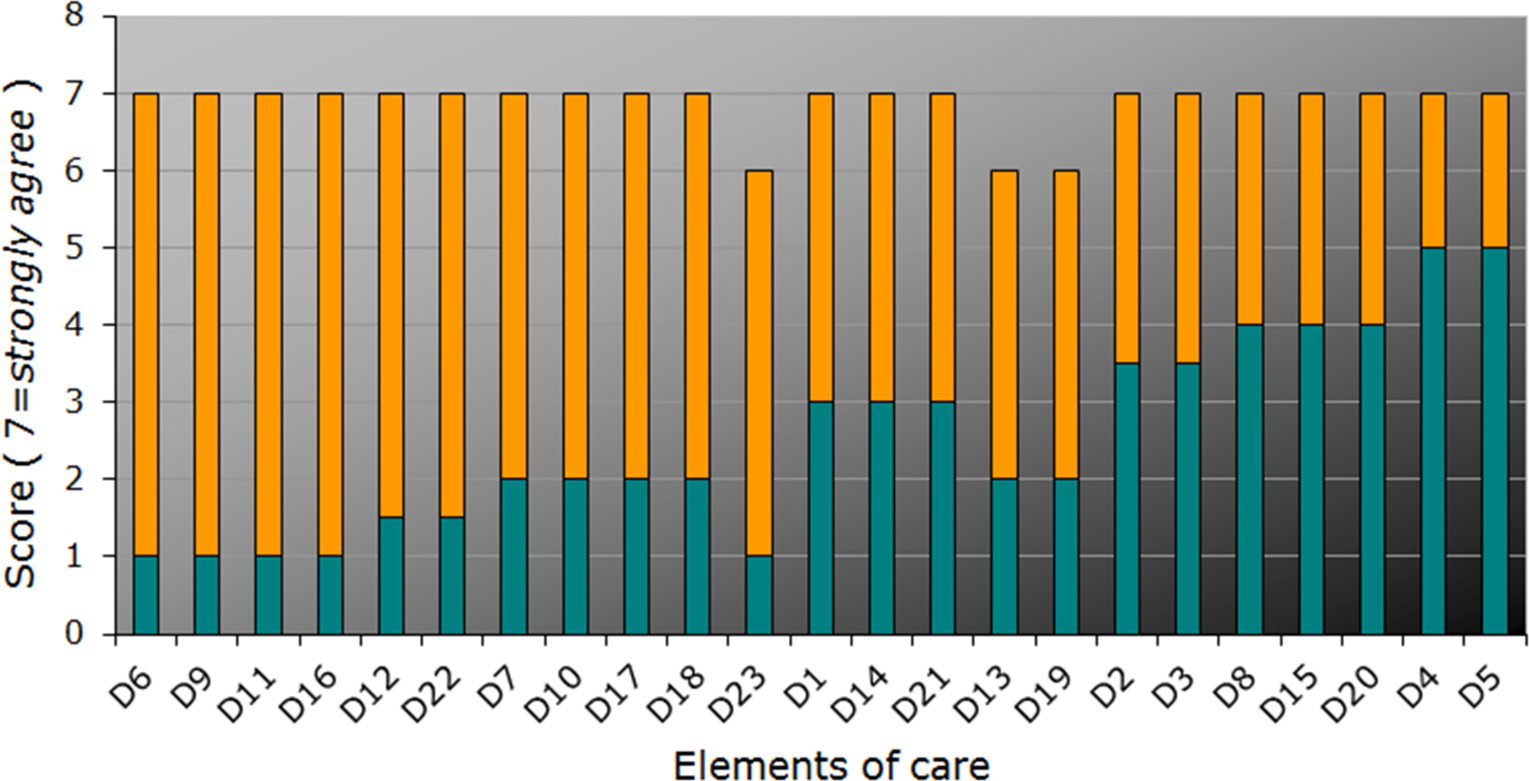
Priority score (*best practice* / “Target”, *full bar height*), current state score (“Actual”, *turquois*), and gap score, *orange*, for items from the “Online-Portal” domain, ordered according to the gap and priority scores (for item descriptions, see Table 2). Each bar represents median scores.

**Fig 3:**
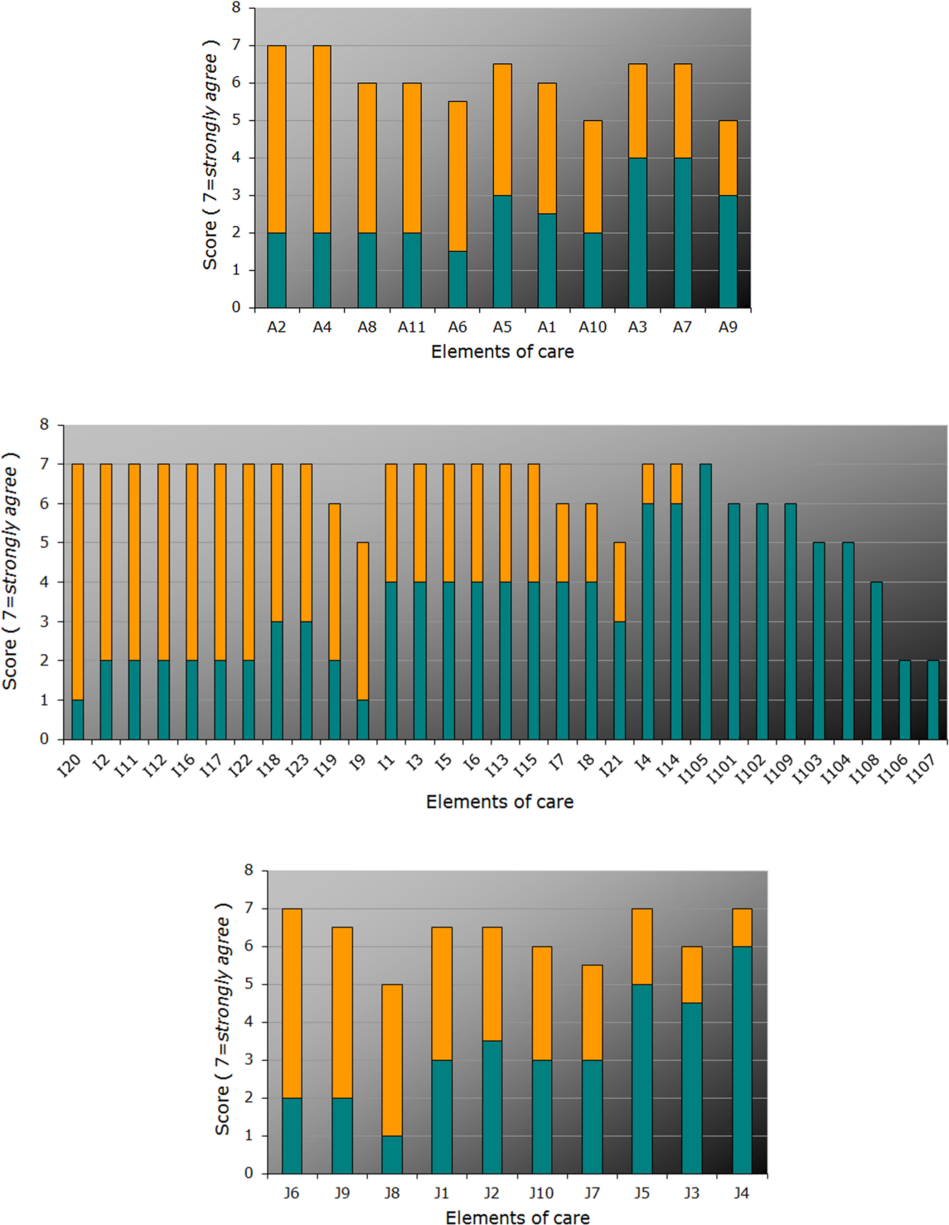
Priority score (*best practice* / “Target”, *full bar height*), current state score (“Actual”, *turquois*), and gap score, *orange*, for items from (**A**) the “Needs of and offers to parents and relatives”, (**B**) “Patient participation”, and (**C**) “Transition programs” domains, ordered according to the gap and priority scores (for item descriptions, see S1–S3 Tables). Each bar represents median scores.

**Fig 4:**
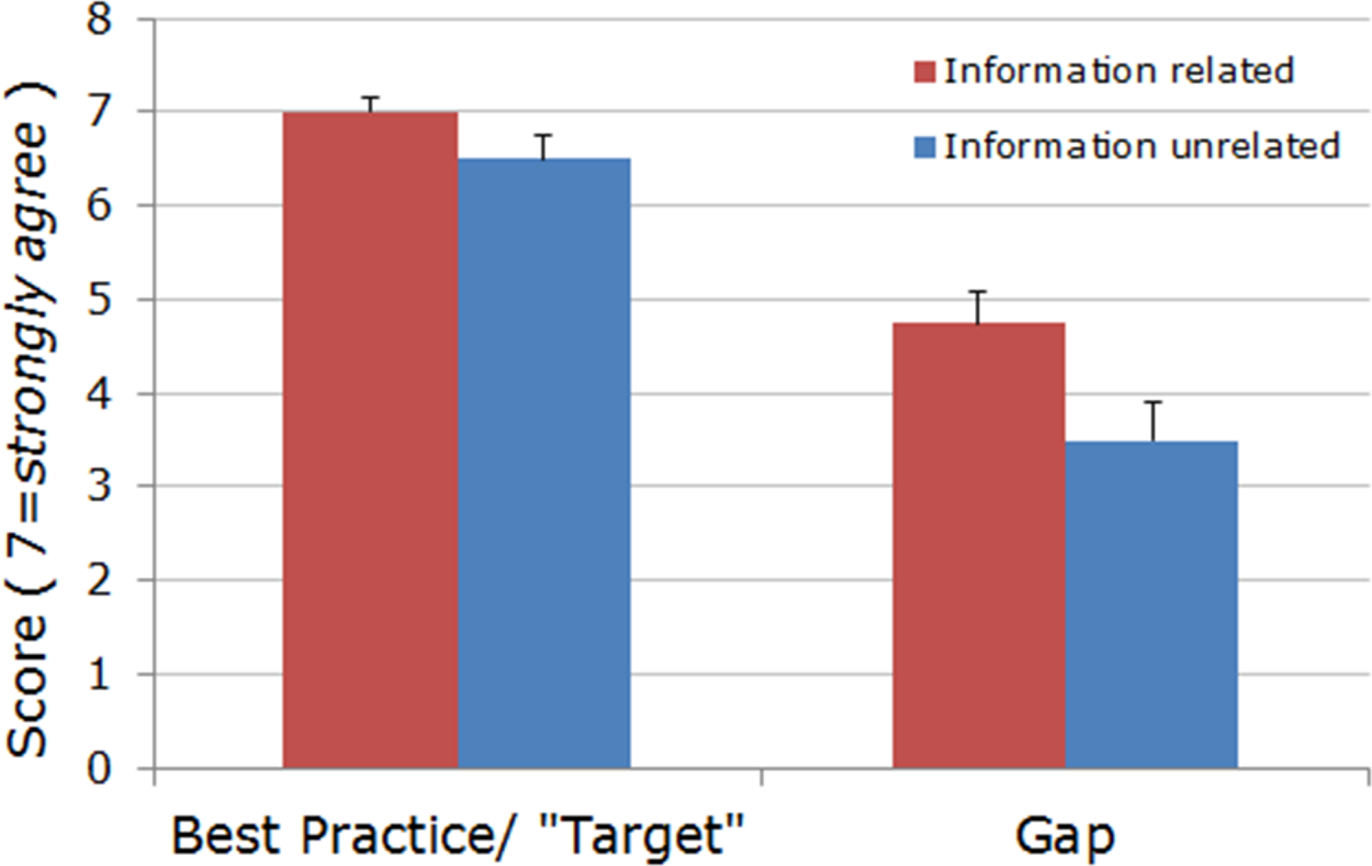
Priority (*best practice* / “Target”, *left*) and gap scores (*right*) for information-related and information-unrelated items from five domains analyzed. Information-related, compared to information-unrelated, items yield both significantly greater priority and gap scores (non-directional Wilcoxon signed-rank *W* test; *P =* 0.008 and *P =* 0.026, respectively). Each bar represents median + 95% confidence interval, CI.

**Fig 5:**
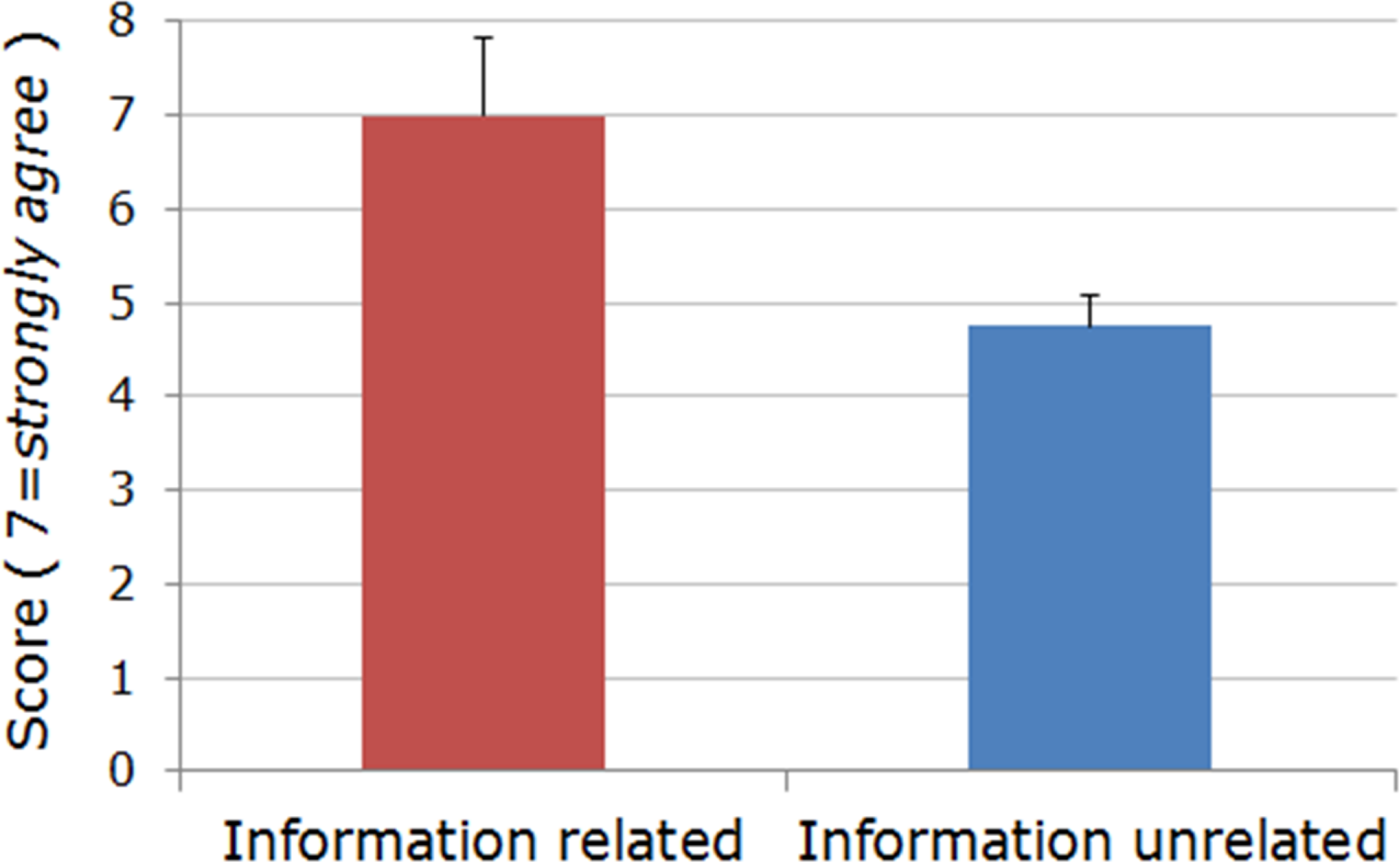
Priority (*best practice* / “Target”) scores for information-related, *left*, and information- unrelated items, *right*, from “Tailored informational offers” domain. Information-related, compared to information-unrelated, items yield significantly greater priority scores (non-directional Wilcoxon signed-rank *W* test; *P =* 0.016). Each bar represents median + 95% CI.

The best practice, experienced current care, and gap scores for the reported domains are shown in Figs 1–5 and Tables 1 and 2 below.

The highest priority scores (7 points for at least top ten items) were obtained for the domains “Online-Portal”, “Patient participation”, and “Tailored informational offers” (Fig 1; Table 1). This characterizes them as particularly important for the *perception as best practice* by affected persons.

The highest gap scores, ranging from 6 to 4 points for the top ten items, were obtained for the domains “Tailored informational offers” (reflecting perceived lack of sufficient disease-related information for affected persons as well as, for example, medical experts and health insurance companies) and “Online-Portal” with only limited information available on specialist clinics and specialized medical doctors (Fig 2; Table 2). Conversely, the lowest gap scores were found with group offers for the MRKHS patients (domain “Transition programs”) and MRKHS self-help days (“Patient participation”). This suggests either satisfaction or rather good solutions in place in the current health care practice. Low gap scores were also obtained for networks for parents (“Needs of and offers to parents and relatives“; Fig 3A), paralleled by lower priority ratings suggesting their minor importance from the point of view of the affected adolescents.

The highest scores for current care, ranging from 6 to 4 points for up to top ten items, occurred for the domains “Online-Portal”, “Patient participation”, and “Transition programs” reflecting quite satisfactory solutions in place in the current health care practice (Figs 2, 3B and 3C). Notably, most of the lowest scores for current care (1 or 2) were obtained for the domain “Tailored informational offers”. Quite a few lowest scores were also found for the domains “Needs of and offers to parents and relatives”, “Online-Portal”, and “Patient participation”, again pointing to the deficits mostly in information availability.

Importantly, when compared across all five domain questionnaires, *information related items* pooled together yielded the highest priority and significantly greater gap scores compared to the remaining, information unrelated items (two-tailed Wilcoxon signed-rank *W* test; *P =* 0.008 and *P =* 0.026 for priority and gap scores, respectively; Fig 4). Moreover, for the domain “Tailored informational offers” alone, information related items received significantly higher priority scores compared with other, information unrelated items (two-tailed Wilcoxon signed-rank *W* test; *P =* 0.016; Fig 5).

It is worth noting that the items were identified during the preceding interviews, that is they had already been prioritized by the respondents and might have been expected to produce a ceiling effect.

## Discussion

To our knowledge, this study is the first to provide patient targeting data that can be expected to meet female adolescents´ needs and preferences with regard to good health care during the transitional phase for those suffering an orphan disease such as MRKHS. So far, the disparities of health care offers as well as the uncertainty regarding the special needs of supportive care have not allowed evidence-based recommendations regarding good transitional care, especially in rare diseases.

The results of 25 in-depth interviews held with affected female patients, mirrored in a final communicative validation and rating, demonstrate that adolescents with MRKHS experience deficits predominantly in availability of information in the present health care services and that they perceive these deficits as crucial barriers to obtaining an appropriate health care. The importance attributed to disease-related tailored information indicates that informational deficits are perceived by the affected patient group as hindering proper access to health care, especially in an orphan disease. It is particularly noteworthy as the items were identified based on the preceding interviews, that is they had already been prioritized by the respondents and might have been expected to produce a ceiling effect. Access to health-related information plays a role for all persons seeking help and care. Previous studies have failed to show the crucial impact of information as factor for gaining access to appropriate health care. To the large part, this may be due to the special situation of persons affected by an orphan disease. The overwhelmingly high scoresattributed to all provisional elements referring to information (content and communication) in the context of an orphan disease, reveal that here, improved information policies are crucial, demanding for institutionalized solutions supported by the health care system.

### Tailored information paralleled by personal offers

At present, access to information seems to be as easy as never before, given the huge variety of internet offers regarding all kinds of information. However, especially patients express uncertainty as to the reliability of these informational offers and still find it difficult to identify the information that best fit their current needs. Evidence is accumulating that patients prefer decision making on health issues in personal communication with medical professionals, especially medical doctors [21]. This includes the validation of information in relation to the personal situation (personalization of information) as well as the discussion of fears, disease-related burdens and health chances, empowering the patient for informed consent. However, in MRKHS as well as other orphan diseases long distances may impair regular personal contacts. The adolescents experiencing this barrier themselves suggest the utilization of the new *e-health related facilities*, as the young generation features technical skills. It is important that launching such communication venues for patients will include data protecting solutions.

### Empowerment for sustainable self-management

In addition to the qualification and the experience of medical professionals, adherence as active contribution of the patients as well as their social, mental and physical conditions are essential to the success of a treatment [22]. Frequently, shame, deficits in information availability and its reliance raise barriers to appropriate communication. Therefore in addition, empowerment of the patient is required: through proper provision of information, the young patient in transition should be qualified to actively engage in his or her treatment, choice of measures, health behavior and health promotion, becoming an *“expert on his or her own disorder”*. The rating outcome mirrors the affected adolescents´ awareness and readiness, calling for a timely and tailored response of the health care system.

Good transitional care is expected to provide patient-centered, age-adjusted, flexible and comprehensive health care offers, taking cultural differences into account, preserving continuity of care by strengthening self-control and self-reliance, and promoting communicative skills [4]. All these steps, however, depend on access to proper comprehendible health and health care system related information. Otherwise, disparities herein will increase variation in health chances.

### Avoiding health inequity

Transition is complicated by further issues such as socioeconomic and regional factors, which increasingly enhance inequity in health chances for young persons in general, as revealed for instance by the BELLA study [23]. These factors interfere additionally with access to specialist care and medical (university) centers [13], which is essential for the patients with orphan disorders. The difficulty in obtaining proper information adds disparity to the interpersonal variation in health literacy due to education and social background.

### Acknowledging patients´ needs–managing their demands

The present findings identify adolescents´ expectations regarding the health care system, particularly stressing the essential role of information. As in such an orphan disease as MRKHS, young people´s health outcome largely depends on proper information, professional solutions seem mandatory. This includes first of all online-offers safeguarding adolescents´ privacy. So far, the German health care system has not anchored such offers in their reimbursement schemes for care in rare diseases. However, current discussions turn to the implementation of centers for rare diseases equipped with dedicated resources [8]. They should include funding of sophisticated e-health solutions for informational offers, “virtual office hours” and augmented staff. The results of this study strongly support such developments: first, as acknowledgment of patients´ needs and second, because in case of rare diseases the supply of accessible, evidence-based medical information can be considered a *prerequisite* for a timely access to proper health care (and thus vital to successful outcomes).

A concurrent reduction of expenses may be expected by avoiding delayed or wrong diagnosis and treatment and by preventing sequelae with regard to physical and mental health through unfavorable patient careers (including the individual pain, loss and public costs). Organizational solutions identified for one condition may prove to be useful for other rare conditions, which would increase the impact of the study.

### Group differences

Low gap scores were found for networks for parents (“Needs and offers for parents and family members“) paralleled by lower priority ratings, suggesting their minor importance as viewed by the affected adolescents. To meet each group´s needs, all interested parties have to be involved that participate in the process of care and support as shown by these examples related to the access to information. Further differences are being elaborated in separate companion publications.

### Limitations

First, the field of rare diseases is a limiting factor in data collection that necessarily restricts the recruitment of large samples. Efforts in several directions were made to overcome basic restrictions for research in orphan diseases as far as possible. Due to the complexity, heterogeneity, individual-centered and evolving nature of transitional care provision and to patients´ and expert insights, e.g., experiences, behavioral aspects, emotional attitudes and preferences are focused, they need being addressed by a qualitative approach. It allows the researchers to explore, for instance, complex attitudes as the experience of disappointment, satisfaction and quality. Personal in-depth interviews, guided by female researchers, seem appropriate for the target patient group, as the subjects were highly individual. The final workshop was held in Open Space Technology [18], which seems appropriate to get insights in preferences and public views.

The ZSFG context has represented a unique advantage that prospective participants out of a considerable number of patients with genital malformations could be approached, within different ranges of age, social background, and patient career, coming from all over the nation and beyond. Sampling hence was oriented in providing diversity as far as possible. The confinement to recruitment within a single though highly specialized institution may fail to obtain regional aspects of other locations. However, the nationwide recruitment managed to include persons of different regional background (e.g., metropolitan areas, rural) as well as varying distances to the center.

Further possible limitations may be due to the national health system background and its impact on the availability of health care elements, especially on the ratings regarding present care. The results concerning best practice seem less affected, as they mirror patients´ and persons´ involved in care and support *needs*.

### Implications for practice

The *clinical implications* of our results should also be delineated. Firstly, the lack of information on the part of those affected, but also their families and attending medical doctors (will be reported separately) affect an effective and efficient supply of services. Rare abnormalities of the female genitals are recognized still often too late. That leads to stressful detours, and partly still inadequate treatment approaches are encountered. Information and networking play a prominent role for those affected. Activities in this direction are attested the best prospects for improving the overall supply capacity of the treatment chain. Finally, technology-based care innovations can lead to an improved situation for rare diseases, for example, by

- creating conditions for new digital technologies, for example, in the form of apps and links as sources for information, support and a personal exchange,
- Internet-based consultations and hotlines (in protected web areas), and
- developing concepts for health literacy and health promotion among young people.

The disparity between experience of care and attribution as best practice, which was detected regarding many elements of care and support, highlights areas of action that affect all domains of care and support involved. The discussion of new concepts and structures for health care in orphan diseases could draw upon such patient-oriented results regarding

1. orphan-disease specific elements, which call for institutionalized reimbursement,
2. elements that are essential for center care and a corresponding network, and
3. elements reflecting patients´ participation in the conception of centers for rare diseases.

## Supporting information

S1 Table ”Needs of and offers to parents and relatives” domain items listed according to their gap and priority scores (including original German version). (DOCX)

S2 Table ”Patient participation” domain items listed according to their gap and priority scores (including original German version). (DOCX)

S3 Table ”Transition programs” domain items ordered according to their gap and priority scores (including original German version). (DOCX)

S4 Table “Tailored information offers” domain items ordered according to their gap and priority scores (original German version; for English version, see main body). (DOCX)

S5 Table ”Online-Portal” domain items ordered according to their gap and priority scores (original German version; for English version, see main body). (DOCX)
