# Supplementary material for: Information ranks highest: Expectations of female adolescents with a rare genital malformation towards health care services
Source: PLoS One. 2017 Apr 20;12(4):e0174031. doi: 10.1371/journal.pone.0174031 (PMC5398506; doi:10.1371/journal.pone.0174031)
Supplement: S1 Table — (DOCX) [file pone.0174031.s001.docx]

**Supporting Information**

**Simoes et al. “Information ranks highest: Expectations of female adolescents with a rare genital malformation towards health care services”**

**S1 Table. ”Needs of and offers to parents and relatives” domain items listed according to their gap and priority scores (including original German version, in *italics*).** The letter (here, A) codes the questionnaire domain and the number (1 to 11), the item’s running position in the questionnaire. Each item had to be ranked using a 7-point scale (1, *strongly disagree*, through 7, *strongly agree*) on two occasions (as to both actual and target, i.e., best practice, state of care).

| **Item** | **Score** | **Item Content** |
| --- | --- | --- |
| A2 | 7 | That there will be information on contact persons in the center or a resprective notice in the (physician's) report, **is / are very important for a good care** \|\| **...is / are implemented in the current care** *[Dass es Informationen über Ansprechpartner_innen im Zentrum bzw. einen Vermerk dazu auf (Arzt‑)berichten gibt,* ***ist / sind sehr wichtig für eine gute Versorgung*** \|\| ***...ist / sind in der Versorgung umgesetzt****]* |
| A4 | 7 | An information flyer on insurance benefits in MRKHS such as regulations as to surgery and travel expenses, compulsory and private health insurance payments, and severely handicapped pass *[Ein Informationsblatt über Versicherungsleistungen bei MRKHS z.B. Regelungen zu Operations- und Fahrtkosten, Leistungen der Gesetzlichen Krankenversicherung, Private Krankenkassen und MRKHS, Schwerbehindertenausweis]* [..] |
| A8 | 6 | That there are consultation hours for parents, *[Dass es Eltern-Sprechstunden gibt,]* [..] |
| A11 | 6 | That there would be permanent counseling offers for parents, during inpatient stays on site, but also in the long-term run (e.g., as to parents' dealing with MRKHS, best possible support of the daughter), *[Dass es durchgängig Beratungsangebote für Eltern gibt, während des stationären Aufenthalts vor Ort, aber auch langfristig (z.B. hinsichtlich eigenem Umgang mit MRKHS, bestmögliche Unterstützung der Tochter) gibt,]* [..] |
| A6 | 5.5 | A parent hotline *[Eine Eltern-Hotline]* [..] |
| A5 | 6.5 | That an offer exists of specific training for parents and a practical advice for daughter support (e.g., „let her go off“, estimating her own expectations, giving room for building identity, support during aftercare), *[Dass es ein Angebot gibt für gezielte Elternschulungen und Praxistipps zur Unterstützung der Tochter (z.B. „Loslassen“ der Tochter, Bewertungen der eigenen Erwartungen, Raum geben für die Identitätsbildung, Unterstützung bei der Nachbehandlung)]* [..] |
| A1 | 6 | That there will be specific offers for parents (forum subdomain for parents at the internet platform), *[Dass es spezielle Angebote für Eltern (Forum-Unterseite für Eltern auf der Internetplattform) gibt,]* [..] |
| A10 | 5 | A possibility of family therapy *[Die Möglichkeit für eine Familientherapie]* [..] |
| A3 | 6.5 | That there will be information on accomodation options in Tübingen, including price and special offers, *[Dass es Informationen zu Unterbringungsmöglichkeiten in Tübingen, deren Kosten und spezielle Angebote gibt]* [..] |
| A7 | 6.5 | That offers exist both for joint and separate counseling, [*Dass es das Angebot zu gemeinsamer oder auch getrennter Beratung gibt*] [..] |
| A9 | 5 | The existence of networks for parents (imparting contacts to other parents, get-together during MRKHS meetings, encouraging to launch such a network by oneself) *[Das Existieren von Eltern-Netzwerken (Kontaktvermittlung zu anderen Eltern, Treffen beim MRKHS- Tag, Anregung selbst ein solches Netzwerk zu organisieren)]* [..] |
