# Supplementary material for: Information ranks highest: Expectations of female adolescents with a rare genital malformation towards health care services
Source: PLoS One. 2017 Apr 20;12(4):e0174031. doi: 10.1371/journal.pone.0174031 (PMC5398506; doi:10.1371/journal.pone.0174031)
Supplement: S2 Table — (DOCX) [file pone.0174031.s002.docx]

**Supporting Information**

**Simoes et al. “Information ranks highest: Expectations of female adolescents with a rare genital malformation towards health care services”**

**S2 Table. ”Patient participation” domain items listed according to their gap and priority scores (including original German version, in *italics*).** The letter (I) codes the questionnaire domain and the number (1 to 23), the item’s running position in the questionnaire. Each item had to be ranked using a 7-point scale (1, *strongly disagree*, through 7, *strongly agree*) on two occasions (as to both actual and target, i.e., best practice, state of care).

| **Item** | **Score** | **Item Content** |
| --- | --- | --- |
| I20 | 7 | That support offers exist in the school and occupational settings, e.g., with occupational choice, academic achievement **is / are very important for a good care** \|\| **...is / are implemented in the current care** *[Dass es Unterstützungs-angebote zur schulischen und beruflichen Situation, z.B. zur Berufswahl, zur Leistung in der Schule* ***ist / sind sehr wichtig für eine gute Versorgung*** \|\| ***...ist / sind in der Versorgung umgesetzt****]* |
| I2 | 7 | That all possible treatment opportunities are communicated during the counseling interview *[Dass im Beratungsgespräch eine Vermittlung von allen möglichen Behandlungsmöglichkeiten erfolgt,]* [..] |
| I11 | 7 | Providing the contact addresses (e.g., of consultant practitioners, psychologists, and self-help groups) in the center *[Die Weitergabe von Adressen von Anlaufstellen (wie z. B. Fachärzten_innen , Psychologen_innen, Selbsthilfegruppen) im Zentrum]* [..] |
| I12 | 7 | That booklets are made available on the MRKHS as rare disease *[Dass Broschüren über die Seltene Erkrankung MRKHS zur Verfügung stehen,]* [..] |
| I16 | 7 | That support offers exist dealing with family and/or financial issues (e.g., relation to parents, low household budget, medical costs) *[Dass es auch Unterstützungsangebote, die familiäre und/oder finanzielle Probleme aufgreifen (z.B. Beziehung zu den Eltern, geringes Budget im Haushalt, Krankheitskosten), gibt,]* [..] |
| I17 | 7 | That support offers exist dealing with social related issues (such as building relationships, mobbing, social exclusion) *[Dass es auch Unterstützungsangebote gibt, die Probleme im sozialen Umgang aufgreifen (z.B. Beziehungsproblemen, Mobbing, Ausgrenzung),]* [..] |
| I22 | 7 | A better fostering of advanced training for pediatricians, general practitioners, and gynecologists *[Eine stärkere Förderung von Weiterbildung bei Kinderärzten_innen, Hausärzten_innen und Gynäkologen_innen]* [..] |
| I18 | 7 | That support offers address also the parents *[Dass sich Unterstützungsangebote auch an Eltern richten,]* [..] |
| I23 | 7 | A systematic evaluation of advanced training measures (control of learning and transfer) *[Eine systematische Evaluation der Weiterbildungen (Lern- und Transferkontrollen)]* [..] |
| I19 | 6 | That support offers address also the partner *[Dass sich Unterstützungsangebote auch an Partner_innen richten,]* [..] |
| I9 | 5 | That interviews are conducted at regular intervals with family members *[Dass regelmäßige Befragungen auch von Angehörigen stattfinden]* [..] |
| I1 | 7 | A full and comprehensible clarification during diagnostic procedures and diagnosis notification *[Eine gründliche und verständliche Aufklärung bei Diagnosestellung bzw. -mitteilung]* [..] |
| I3 | 7 | That the affected individual’s own report (also of underage persons) will take place (e.g., during physician-patient communication, with the nurse while in the inpatient care*) [Dass eine persönliche Ansprache der Betroffenen (auch bei Minderjährigen) erfolgt (z.B. im Arzt-Patienten-Gespräch, auf Station beim Gespräch mit der Krankenschwester)]* [..] |
| I5 | 7 | That there is a possibility, also for an underage person on her own to get an appointment in the center for a confidential (“four-eyes”) consultation *[Dass die Möglichkeit besteht, sich auch als Minderjährige alleine zu einem Gespräch „unter vier Augen“ und in der Sprechstunde des Zentrums vorzustellen,]* [..] |
| I6 | 7 | A systematic recording and processing of patient complaints (complaint management) *[Ein systematisches Erfassen und Bearbeiten von Patientenbeschwerden (Beschwerdemanagement)]* [..] |
| I13 | 7 | That the internet addresses of MRKHS related websites are listed on the social networks and information platforms *[Dass Internetadressen von MRKHS-spezifischen Seiten in sozialen Netzwerken und Informationsplattformen zur Verfügung stehen,]* [..] |
| I15 | 7 | That MRKHS self-help groups exist *[Dass es MRKHS-Selbsthilfegruppen gibt,]* [..] |
| I7 | 6 | That there is a collecting box for feedback, requests, and concerns (feedback box) *[Dass es einen Sammelkasten für Rückmeldungen, Anliegen (Feedbackbox) gibt,]* [..] |
| I8 | 6 | That interviews are conducted at regular intervals as to patient satisfaction *[Dass regelmäßige Befragungen zur Patientenzufriedenheit stattfinden,]* [..] |
| I21 | 5 | That at all times if required, there is a psychological counseling offer available *[Dass jederzeit das Angebot besteht, falls gewünscht, psychologische Beratung zu erhalten,]* [..] |
| I4 | 7 | That if required, there is a possibility of a confidential („four-eyes“) consultation *[Dass die Möglichkeit zu einem Gespräch “unter vier Augen”, falls gewünscht, sichergestellt ist,]* [..] |
| I14 | 7 | That MRKHS self-help days exist *[Dass es einen MRKHS-Selbsthilfetag gibt,]* [..] |
