# Supplementary material for: Information ranks highest: Expectations of female adolescents with a rare genital malformation towards health care services
Source: PLoS One. 2017 Apr 20;12(4):e0174031. doi: 10.1371/journal.pone.0174031 (PMC5398506; doi:10.1371/journal.pone.0174031)
Supplement: S3 Table — (DOCX) [file pone.0174031.s003.docx]

**Supporting Information**

**Simoes et al. “Information ranks highest: Expectations of female adolescents with a rare genital malformation towards health care services”**

**S3 Table. ”Transition programs” domain items ordered according to their gap and priority scores (including original German version, in *italics*).** The letter (J) codes the questionnaire domain and the number (1 to 10), the item’s running position in the questionnaire. Each item had to be ranked using a 7-point scale (1, *strongly disagree*, through 7, *strongly agree*) on two occasions (as to both actual and target, i.e., best practice, state of care).

| **Item** | **Score** | **Item Content** |
| --- | --- | --- |
| J6 | 7 | Assistance with search for psychological support (enhanced transfer to the psychotherapist) **is / are very important for a good care** \|\| **...is / are implemented in the current care** *[Hilfe bei der Suche nach psychologischer Unterstützung (erleichterter Übergang zum Therapeuten / zur Therapeutin)* ***ist / sind sehr wichtig für eine gute Versorgung*** *\|\|* ***...ist / sind in der Versorgung umgesetzt***] |
| J9 | 6.5 | That the hospital (center) has a dedicated space / room, in which MRKHS patients can get together *[Dass es bestimmte Bereiche / Zimmer im Krankenhaus (Zentrum) gibt, wo Patientinnen mit MRKHS zusammen sind,]* [..] |
| J8 | 5 | Group offers for significant others (e.g., family therapy) *[Gruppenangebote für soziale Bezugspersonen (z.B. Familientherapie)]* [..] |
| J1 | 6.5 | The multidisciplinary (transprofessional) development of a transition roadmap (a written plan for the progression of care) *[Das multidisziplinäre (berufsgruppenübergreifende) Erstellen eines Transitionsplans (schriftlicher Plan für den Versorgungsablauf)]* [..] |
| J2 | 6.5 | Participation of affected persons in the development of a transition roadmap *[Die Beteiligung der Betroffenen an der Erstellung eines Transitionsplans]* [..] |
| J10 | 6 | That the center has a dedicated space with youth friendly environment (room interior, games, reading material, etc.) *[Dass es bestimmte Bereiche im Zentrum gibt, die eine spezielle jugend-freundliche Ausstattung haben (Raumgestaltung, Spiele, Lesestoff u.a.),]* [..] |
| J7 | 5.5 | Specific offers for significant others (e.g., special consultation hours for parents) *[Einzelangebote für soziale Bezugspersonen (z.B. Spezialsprechstunde für Eltern)]* [..] |
| J5 | 7 | Specific offers for affected persons (e.g., special practitioner consultation hours) *[Einzelangebote für Betroffene (bspw. Ärztliche Spezialsprechstunden)]* [..] |
| J3 | 6 | A permanent attachment person for the whole transition period (e.g., nurse, „guardian physician“, health visitor) *[Eine konstante Bezugsperson für den gesamten Transitionsverlauf (z.B. Krankenschwester, „Bezugsärztin“, Sozialarbeiter_in),]* [..] |
| J4 | 7 | Group offers for affected persons (e.g., self-help days) *[Gruppenangebote für Betroffene (z.B. Selbsthilfetage)]* [..] |
