# Supplementary material for: Information ranks highest: Expectations of female adolescents with a rare genital malformation towards health care services
Source: PLoS One. 2017 Apr 20;12(4):e0174031. doi: 10.1371/journal.pone.0174031 (PMC5398506; doi:10.1371/journal.pone.0174031)
Supplement: S4 Table — (DOCX) [file pone.0174031.s004.docx]

**Supporting Information**

**Simoes et al. “Information ranks highest: Expectations of female adolescents with a rare genital malformation towards health care services”**

**S4 Table. “Tailored information offers” domain items ordered according to their gap and priority scores (original German version; for English version, see main body).** The letter (F) codes the questionnaire domain and the number (1 to 18), the item’s running position in the questionnaire. Each item had to be ranked using a 7-point scale (1, *strongly disagree*, through 7, *strongly agree*) on two occasions (as to both actual and target, i.e., best practice, state of care).

| **Item** | **Score** | **Item Content** |
| --- | --- | --- |
| F1 | 7 | Bebildertes Informationsmaterial (z.B. zu Operationsmethoden) **ist / sind sehr wichtig für eine gute Versorgung** \|\| **...ist / sind in der Versorgung umgesetzt** |
| F2 | 7 | Ein medizinisches Informationsblatt zu MRKHS für Nachbehandler_innen mit Ansprechpartner_innen im Zentrum [..] |
| F3 | 7 | Informationen über Zentren, Ansprechpartner_innen usw. [..] |
| F4 | 7 | Ein Informationsblatt zu Angeboten der psychologischen Beratung und Therapie (mit z.B. speziellen Therapeut_innen-Adressen) [..] |
| F7 | 7 | Informationen über Selbsthilfenetzwerke und Foren [..] |
| F9 | 7 | Ein Flyer zum Krankheitsbild (z.B. Was ist MRKHS?) [..] |
| F10 | 7 | Ein Informationsblatt über Versicherungsleistungen bei MRKHS (z.B. Regelungen zu Operations- und Fahrtkosten, Leistungen der Gesetzlichen Krankenversicherung, Private Krankenkassen und MRKHS-Schwerbehindertenausweis) [..] |
| F11 | 7 | Für medizinische Expert_innen: ein MRKHS-Flyer mit Fachinformationen (z.B. zum Verlauf: Diagnose – OP – Nachsorge, Fachzentren und Ansprechpersonen; Hilfestellung für Rezeptierung von z.B. Creme und Miederhöschen [..] |
| F12 | 7 | Für medizinische Expert_innen: ein MRKHS- Flyer mit Fachinformation zur Differentialdiagnose (z.B. bei Ausbleiben der Periode) [..] |
| F13 | 7 | Ein Flyer für Krankenkassen zum Krankheitsbild MRKHS und den Therapien (z.B. Operationsformen, Rezeptieren von mehreren Miederhöschen, Phantomen, Cremes) [..] |
| F14 | 7 | Für Krankenkassen: ein Case Management Schulungsangebot (z.B. „Expert_innen für Seltene Erkrankung“ bei den Krankenkassen intern) [..] |
| F16 | 7 | Die Behandlung von MRKHS und anderen genitalen Fehlbildungen z.B. im (Biologie-)Unterricht (Lehrplanerweiterung) [..] |
| F8 | 6.5 | Ein FAQ Flyer (zu allen Fragen rund um MRKHS) [..] |
| F15 | 6 | Für den Gemeinsamen Bundesausschuss: Informationen, die Sonderrege-lungen begründen für Patien_innen mit Seltenen Erkrankungen (hinsichtlich z.B. Budget für Spezialambulanz, Qualitätsgesicherte Zentren, erweiterte hausärztliche Versorgung, Hilfsmittelverzeichnis) [..] |
| F5 | 5.5 | Ein Informationsblatt für Supervisionsangebote, Coaching [..] |
| F6 | 5 | Informationen zu Ausbildungs-, Weiterbildungs-, Karriereberatung [..] |
| F17 | 5 | Ein Schulungsangebot durch das Zentrum für interessierte Lehrer_innen zu MRKHS und seltenen genitalen Fehlbildungen und deren didaktische Aufarbeitung im Schulunterricht] [..] |
| F18 | 4 | Eine Weiterqualifikation für Vertrauenslehrer_in zu seltenen Erkrankungen, speziell zu MRKHS] [..] |
