# Supplementary material for: Information ranks highest: Expectations of female adolescents with a rare genital malformation towards health care services
Source: PLoS One. 2017 Apr 20;12(4):e0174031. doi: 10.1371/journal.pone.0174031 (PMC5398506; doi:10.1371/journal.pone.0174031)
Supplement: S5 Table — (DOCX) [file pone.0174031.s005.docx]

**Supporting Information**

**Simoes et al. “Information ranks highest: Expectations of female adolescents with a rare genital malformation towards health care services”**

**S5 Table. ”Online-Portal” domain items ordered according to their gap and priority scores (original German version; for English version, see main body).** The letter (D) codes the questionnaire domain and the number (1 to 23), the item’s running position in the questionnaire. Each item had to be ranked using a 7-point scale (1, *strongly disagree*, through 7, *strongly agree*) on two occasions (as to both actual and target, i.e., best practice, state of care).

| **Item** | **Score** | **Item Content** |
| --- | --- | --- |
| D1 | 7 | Informationsseiten auf der Internetplattform des Zentrums, die zugeschnitten sind auf die jeweiligen speziellen Fragen und Anliegen für jede involvierte Gruppe - Betroffene, Eltern, Geschwister, Partner_innen, niedergelassene Ärzt_innen u.a. **sind / ist sehr wichtig für eine gute Versorgung** \|\| **…sind / ist in der Versorgung umgesetzt** |
| D2 | 7 | Dass die Sicherheit der (Forums-)Teilnehmenden durch teils offene und teils geschützte Bereiche auf der Internetplattform des Zentrums gewährleistet ist, [..] |
| D3 | 7 | Geschlossene (geschützte) Fragenportale und Diskussionsforen für einzelne Gruppen [..] |
| D4 | 7 | Dass die Internetplattform des Zentrums Antworten zu häufig gestellten Fragen (FAQ) anbietet, [..] |
| D5 | 7 | Eingehende Informationen zu MRKHS (auch zu Hintergründen) auf der Internetplattform des Zentrums [..] |
| D6 | 7 | Informationen auf der Internetplattform des Zentrums zu anderen Kliniken und niedergelassenen Ärzt_innen, die sich auf MRKHS spezialisiert haben (und mit der Nennung einverstanden sind) [..] |
| D7 | 7 | Informationen zu Kontaktadressen auf der Internetplattform des Zentrums [..] |
| D8 | 7 | Informationen zu Therapiemöglichkeiten, speziell Operationsmethoden auf der Internetplattform des Zentrums [..] |
| D9 | 7 | Informationen zum Stand der Forschung (z.B. Gebärmuttertransplantation) [..] |
| D10 | 7 | Informationen zur Adoption auf der Internetplattform des Zentrums [..] |
| D11 | 7 | Informationen bezüglich Links zu geeigneten Fernsehbeiträgen auf der Internetplattform des Zentrums [..] |
| D12 | 7 | Dass Informationsbroschüren und Flyer als pdf von der Internetplattform des Zentrums heruntergeladen werden können (z.B. als Unterstützung für Hausärzt_innen) [..] |
| D14 | 7 | Links von Informationsplattformen untereinander bzw. über das Zentrum [..] |
| D15 | 7 | Dass die Internetplattform des Zentrums schnell im Internet auffindbar, überschaubar, zugänglich und allgemein verständlich ist [..] |
| D16 | 7 | Dass die Informationsplattform des Zentrums durch eine_n hauptamtliche_n Administrator_in bearbeitet wird, so dass die konstante Aktualisierung sichergestellt ist (z.B. neueste Forschungsergebnisse z.B. zur Uterustransplantation) [..] |
| D17 | 7 | Dass die MRKHS-Foren moderiert und kontrolliert sind, [..] |
| D18 | 7 | Dass in kurzen Zeitabständen und zuverlässig von Expert_innen auf Fragen, die über die Internetplattform des Zentrums gestellt werden, reagiert wird, [..] |
| D20 | 7 | Aktuelle Informationen auch zu Treffen von Selbsthilfegruppen auf der Internetplattform des Zentrums [..] |
| D21 | 7 | Aktuelle Informationen auch zu Tagungen und Weiterqualifizierungsmaß-nahmen, bezüglich MRKHS auf der Internetplattform des Zentrums [..] |
| D22 | 7 | Links zu mitwirkenden Krankenkassen zu Leistungsinformationen (z.B. Finanzierungsanfragen zu weiteren Miederhöschen, Phantomen) auf der Internetplattform des Zentrums [..] |
| D13 | 6 | Dass die Informationsplattform des Zentrums so strukturiert ist, dass es einen zentralen Bereich und spezifische Anteile z.B. zu Spezialthemen, für unterschiedliche Gruppen, gibt, [..] |
| D19 | 6 | Dass beauftragte Expert_innen des Zentrums über ein entsprechendes Zeitbudget zur Mitarbeit in der Informationsplattform verfügen, [..] |
| D23 | 6 | Ein Informationsblatt über Versicherungsleistungen bei MRKHS z.B. Regelungen zu Operations- und Fahrtkosten, Leistungen der Gesetzlichen Krankenversicherung, Private Krankenkassen und MRKHS, Schwerbehindertenausweis [..] |
